# Temporal expression pattern of genes during the period of sex differentiation in human embryonic gonads

**DOI:** 10.1038/s41598-017-15931-3

**Published:** 2017-11-21

**Authors:** Linn S. Mamsen, Emil H. Ernst, Rehannah Borup, Agnete Larsen, Rasmus H. Olesen, Erik Ernst, Richard A. Anderson, Stine G. Kristensen, Claus Y. Andersen

**Affiliations:** 1 Laboratory of Reproductive Biology, Section 5712, The Juliane Marie Centre for Women, Children and Reproduction, University Hospital of Copenhagen, University of Copenhagen, Rigshospitalet, Blegdamsvej 9, 2100 Copenhagen, Denmark; 2 Department of Biomedicine - Pharmacology, Aarhus University, Bartholins Allé 6, 8000 Aarhus C, Denmark; 3 Randers Regional Hospital, 8930 Randers, NØ Denmark; 4 Microarray Center of Righshospitalet, Genomic Medicine, University Hospital of Copenhagen, University of Copenhagen, Rigshospitalet, Blegdamsvej 9, 2100 Copenhagen, Denmark; 5 Functional Genomics and Reproductive Health Group, Center for Chromosome Stability, Department of Cellular and Molecular Medicine, University of Copenhagen, Blegdamsvej 3B, Copenhagen, Denmark; 6 Department of Obstetrics and Gynaecology, University Hospital of Aarhus, Skejby Sygehus, 8200 Aarhus N, Denmark; 7 MRC Centre for Reproductive Health, University of Edinburgh, 47 Little France Crescent, EH16 4TJ Edinburgh, United Kingdom

## Abstract

The precise timing and sequence of changes in expression of key genes and proteins during human sex-differentiation and onset of steroidogenesis was evaluated by whole-genome expression in 67 first trimester human embryonic and fetal ovaries and testis and confirmed by qPCR and immunohistochemistry (IHC). *SRY/SOX9* expression initiated in testis around day 40 pc, followed by initiation of *AMH* and steroidogenic genes required for androgen production at day 53 pc. In ovaries, gene expression of *RSPO1, LIN28*, *FOXL2, WNT2B*, and *ETV5*, were significantly higher than in testis, whereas *GLI1* was significantly higher in testis than ovaries. Gene expression was confirmed by IHC for GAGE, SOX9, AMH, CYP17A1, LIN28, WNT2B, ETV5 and GLI1. Gene expression was not associated with the maternal smoking habits. Collectively, a precise temporal determination of changes in expression of key genes involved in human sex-differentiation is defined, with identification of new genes of potential importance.

## Introduction

Gonadal sex-differentiation has been extensively studied in animal models but the precise timing of genetic events leading to proper development and sex-differentiation in humans is still uncertain. Significant information has been obtained in mice studies and similar mechanisms during sex differentiation are seen^1^. However, increasing evidence suggest that inter-species differences exist in the mechanisms of sex differentiation and that studies of human material is essential to verify results from other species^2–7^. A number of studies have suggested that fetal exposure to environmental pollutants, including maternal cigarette smoking, compromise germ cell number and potentially future fertility^8–12^, highlighting the events underlying gonadal differentiation as essential for understanding how external factors may affect gonadal development. In murine studies, several genes, including the Wilms tumor suppressor gene (*WT1)*, steroidogenic factor 1 (*SF1*) and the Lim homeobox protein gene *(LIM1)* are necessary for the development of the bi-potential gonad prior to sex-differentiation^13,14^. Expression of these genes in human gonads is not clarified.

It was long held that sex-differentiation into testis required the expression of specific genes, while their absence would result in development of an ovary. It is now clear that gonadal sex is determined by antagonistic interactions between ovarian and testicular pathways^15,16^. In humans, the gonads are populated by primordial germ cells (PGCs), deriving from the yolk sac wall early in week five post conception (pc)^17,18^, with sex differentiation initiating around week six pc^18^. Somatic cell lines derive from the mesonephros and in females also from the ovarian surface epithelium^19,20^. In mammals, the crucial step towards differentiation into testis depends on the activation of the sex determining region on the Y chromosome (*SRY*), in which mutations lead to sex reversal^2,21–24^. In rodents, expression of *Sry* initiates a cascade of downstream signalling through the direct regulation of *Sry*-related HMG-Box gene 9 (*Sox9)*. This promotes differentiation of the supporting cell precursors into Sertoli cells synthesising anti-Müllerian hormone (AMH)^25–27^. Several of the genes known to be essential for sex determination are highly conserved in mammals including human and mouse: WT-1^28–30^, SF-1^30–32^, SOX9^2,3,33^, AMH^34,35^, whereas mutations for instance in the *DAX1* gene lead to adrenal hypoplasia and hypogonadotropic hypogonadism in humans^36^, with no effect on gonadal development and spermatogenesis in murine *Dax1* null mutation^37^. It has been suggested that the mutant mouse may not be a complete null mutant or that *Dax1* acts differently in mouse and human^38^. Inter-species differences have also been seen in the *SRY* gene. Human *SRY* transcripts and protein are persistent in low levels throughout the embryonic period^3^, whereas in the mouse *Sry* is expressed in a peak initiating differentiation^39^. Further neither human SRY or SOX9 transgenes are able to substitute for their mouse paralogs in transgene experiments^4^, supporting the inter-species differences. The *SRY*-related HMG-Box gene 17 (*SOX17)* has been described as an important regulator of germ cell specification in the human gonad only^5,40^. The pluripotent transcription factor *OCT4* have recently been reported to be essential for blastocyst formation in humans – not in mice, suggesting an earlier and different role of OCT4 in human blastocysts compared to mice^7^.

In human testis, Leydig cell differentiation is dependent of the establishment of the sex cords and the first Leydig cells can be recognized at the end of week 9 pc^18,41^ simultaneous with the first testosterone production^42^, suggesting that the crucial events initiating steroidogenesis has taken place at this time, though the exact timing of the steroidogenic initiation is unknown. In human females, absence of *SRY* alongside expression of ovary-determining-genes such as Wnt Family Member 4 (*WNT4)*, roof plate-specific spondin-1 (*RSPO1)* and forkhead box L2 (*FOXL2)* appear important for ovarian differentiation^43^. However, most of the experimental basis derives from mouse knockout models, with supporting information from genetic analysis of individuals with disorders of sexual development^43,44^.

Comprehensive analysis of gene expression in normal human gonads from the embryonic and early fetal stage has been limited^44–46^. No studies has yet addressed the precise timing and sequence of changes in expression of key genes and proteins at the time of sex differentiation and related that to subsequent events in germ cell and somatic cell maturation.

The aim of the present study was to analyse whole-genome gene expression in human male and female gonads aged 40 to 73 days pc. This period covers sex differentiation, testicular cord formation, and the testicular onset of steroidogenesis, with contemporaneous changes in the ovary.

## Materials and Methods

A total of 67 first-trimester human embryonic and fetal gonads aged 40–73 days pc were included. Global gene expression was performed in 46 gonads (27 males, 19 females) by HTA-2.0 microarray analysis (Supp. Table 1). In 13 cases two gonads from the same embryo were obtained; in these cases one gonad was included in the microarray analysis while the other was used for qPCR validation. Immunohistochemical (IHC) analyses were performed at 8 embryonic and fetal gonads of both sexes.

### Participating women

All participants were healthy women aged 18–47 years (mean ± SEM, 26.0 ± 1.0). All samples were obtained from legal elective abortions before gestational week 12 and all appeared morphologically normal. Exclusion criteria included age below 18 years, chronic disease, requiring an interpreter, and pregnancies with known disorders. All participants received oral and written information and gave their informed consent. Participants answered a questionnaire concerning their lifestyle during the pregnancy, including smoking and drinking habits. (Supp. Table 2). All methods were performed in accordance with the relevant guidelines and regulations and was approved by ‘The Scientific Ethical Committee for the Capital Region’ [KF (01) 258206].

### Human embryonic and fetal tissues

Samples were obtained following surgical abortion at Skejby University Hospital. One ovary (73 days pc) was obtained from the archives at the University of Copenhagen. Embryonic and fetal age was determined by crown-rump length measured by ultrasound *in utero*. The genetic sex were determined by gonadal morphology and confirmed by PCR^47^.

### Tissue processing and RNA extraction

Within minutes after the surgical procedure the aborted tissue was dissected under a stereomicroscope and the gonads were isolated from mesonephros. Gonads for IHC were fixed in Bouins solution and processed for histology. Gonads for gene analysis were incubated in RNAlater® (Sigma-Aldrich, Copenhagen, Denmark) for 5 min and stored at −80 °C. Prior to RNA isolation the samples were homogenized in a TissueLyser II at 4 °C (Qiagen, Copenhagen, Denmark) in 1.0 mL TRI Reagent (Sigma-Aldrich) for 2 × 30 seconds at 15 Hz, using 0.3 mm. stainless steel beads. Each sample was further homogenized by adding 200 µl chloroform followed by vigorously shaking for 15 seconds followed by incubation at room temperature for 2–3 min. Samples were centrifuged through MaxTract High Density tubes (cat. No.: 129056, Qiagen) at 12,000 × *g* for 15 min at 4 °C. The further processing of the RNA containing supernatants were processed according to^48^. Median RIN was 9.3, range (2.4–10.0).

### Microarray analysis

RNA samples were amplified and labelled using the Ovation Pico WTA v.2 RNA Amplification System according to the manufacturer’s instructions (Nugen, San Carlos, CA, USA). First strand cDNA was prepared from 50 ng of total RNA using a unique first strand DNA/RNA chimeric primer mix and reverse transcriptase (RT). Fragmentation of the mRNA within the cDNA/mRNA complex created priming sites for DNA polymerase to synthesize a second cDNA strand, which was used in the following single primer isothermal amplification (SPIA) step, in which the process of SPIA DNA/RNA primer binding, DNA replication, strand displacement and RNA cleavage was repeated to produce cDNA. Single stand cDNA was fragmented and biotin-labelled using the Encore Biotin Module (Nugen, San Carlos, CA, USA) before hybridization to the Human Transcriptome Array 2.0 GeneChip® (HTA 2.0) (Affymetrix, Santa Clara, CA, USA). The arrays were washed and stained with phycoerythrin conjugated streptavidin (SAPE) using the Affymetrix Fluidics Station® 450, and further scanned in the Affymetrix GeneArray® 2500 scanner to generate fluorescent images, as described in the Affymetrix GeneChip® protocol. Cell intensity files (CEL files) were generated in the GeneChip® Command Console® Software (AGCC) (Affymetrix). The data was normalized and modelled using the RMA (Robust Multichip Average). The resulting expression matrix was pre-filtered according to Bourgon and colleagues^49^ by independent filtering of log2 values above 2.1 in one of the sexes in order to allow detection of sex dimorphic gene expressions and remove genes expressed below background. Only functionally annotated genes were included in the further analysis. Log2 expression between 2.1 and 5 was defined as genes expressed in a compartment of cells and log2 above 5 as genes being expressed in either all cells or abundantly in a subset of cells.

### Quantitative real-time PCR analysis

Total RNA was extracted from whole homogenized embryonic and fetal gonads using TRI Reagent (Sigma-Aldrich,) and converted to first-strand cDNA by the use of the High Capacity cDNA Reverse Transcriptase Kit (Applied Biosystems, Carlsbad, CA, USA) according to the manufactures instructions. Samples were kept on ice at all times. Gene expression was determined by quantitative real-time PCR with the TaqMan® detection system as previously described^48^, using probes ids: SRY, #Hs00976796_s1, SOX-9, #Hs01001343_g1; CYP11A1, #Hs00167984_m1; STAR, #Hs00986559_g1; LIN28A, #Hs00702808_s1. Human glyceraldehyde 3-phosphatdehydrogenase (GAPDH) was used as endogen control (probe id. No.: 433764 F). All samples were normalized to GAPDH and the relative expression was quantified according to the Comparative CT Method^50^.

### Germ cell density

The germ cell density (i.e. number of germ cells per mm^3^) in embryonic and fetal gonads was calculated from our previously published data on gonadal germ cell numbers^8,9^. The germ cell densities were measured in gonads, which had or had not been exposed to maternal cigarette smoking.

### Immunohistochemistry (IHC)

Five μm serial sections were de-paraffinated in xylene, rehydrated in ethanol before antigen retrieval in Tris-egtazic acid (TEG-buffer) (10 mM Tris, 0.5 mM egtazic acid, pH 9) or citrate buffer (10 mM sodium citrate, pH 6) for 20 minutes. Endogen activity was inhibited with 1.5% peroxidase for 10 minutes, followed by one hour inhibition of unspecific binding with 1% bovine serum albumin (BSA) (Sigma Aldrich). Sections were incubated with primary antibodies for one hour at room temperature (Table 1). GLI1 and ETV5 were incubated over night at 4 °C. Secondary antibodies used were rabbit-anti-mouse-HRP (Dako, Glostrup, Denmark, 1:100) and goat-anti-rabbit (Zymed, California, US 1:100) and visualised with 3.3′-diaminobenzidine tetrahydrochloride (DAB + , Dako, 1:100).

**Table 1:** Antibodies, provider, catalogue number, antibody-specie, antigen retrieval, dilution.

Antibody	Supplier	Cat. no.	Species	Retrival	Dilution
GAGE	Transduction laboratories^a^	877-232-8995	Mouse	Citrate, pH: 6	1:200
LIN28	Abcam^b^	Ab-46020	Rabbit	Citrate, pH: 6	1:500
AMH	Ansh Laboratories^c^	AMH-72	Mouse	Citrate, pH: 6	1:200
SOX9	Chemicon^d^	Ab-5535	Rabbit	Citrate, pH: 6	1:500
CYP17A1	Santa Cruz Biotechnologies^e^	Sc-374244	Mouse	TEG, pH: 9	1:100
WNT2B	Abcam^b^	Ab-150612	Rabbit	Citrate, pH: 6	1:40
GLI1	Santa Cruz Biotechnologies^e^	Sc-515751	Mouse	TEG, pH: 9	1:100
ETV5	Santa Cruz Biotechnologies^e^	Sc-100941	Mouse	TEG, pH: 9	1:50

^a^BD Bioscience, Albertslund, Denmark.

^b^Cambridge, UK.

^c^Texas, US.

^d^Illinois, US.

^e^Heidelberg, Germany.

### Statistical methods

Statistical analysis was performed using GraphPad Prism 6.07 program (GraphPad Software, Inc., CA, USA) and the R program together with RStudio, version 0.99.473 (R software, Boston, Massachusetts, USA). Significance level was defined as a probability lower than 0.05 (p < 0.05). For each gene of interest data were analysed as fitting a linear or quadric expression pattern, for males and females separately. Differences in gene expression between sexes were further analysed with a linear or quadric model according to the results from the initial test. A Spearman’s rank test was used to evaluate whether gene expressions correlated with age. To compare characteristics from smoking versus non-smoking women, an unpaired parametric t-test was used when the parameters were normally distributed (e.g. age and BMI); when the parameters were not normally distributed, an unpaired non-parametric Mann-Whitney test was used (e.g. smoking).

## Results

A total 46 human fetal gonads (27 males and 19 females) aged 40–67 days (mean ± SEM, 53.1 ± 1.1) were evaluated by global gene expression analysis. Further, 13 gonads were included for qPCR validation and 8 gonads were included for IHC.

### Array gene expression

A total of 70,500 transcripts were analysed, including control, coding, non-coding and normalization genes. A total of 67,525 transcribed clusters were found, with 26,800 coding genes of which annotated genes extracted via the Qlucore Omics Explorer software (Qlucore.com). A sex dimorphic expression was detected in 319 genes (fold change >2.0, false discovery rate p-value < 0.05) (Supp. Figure 1). A total of 32 new and known genes associated with early gonadal development and sex-differentiation, including genes not previously described, were selected based on the expression level for further analysis and mapping.

### Expression of genes determining development of the bi-potential gonad

**Figure 1:**
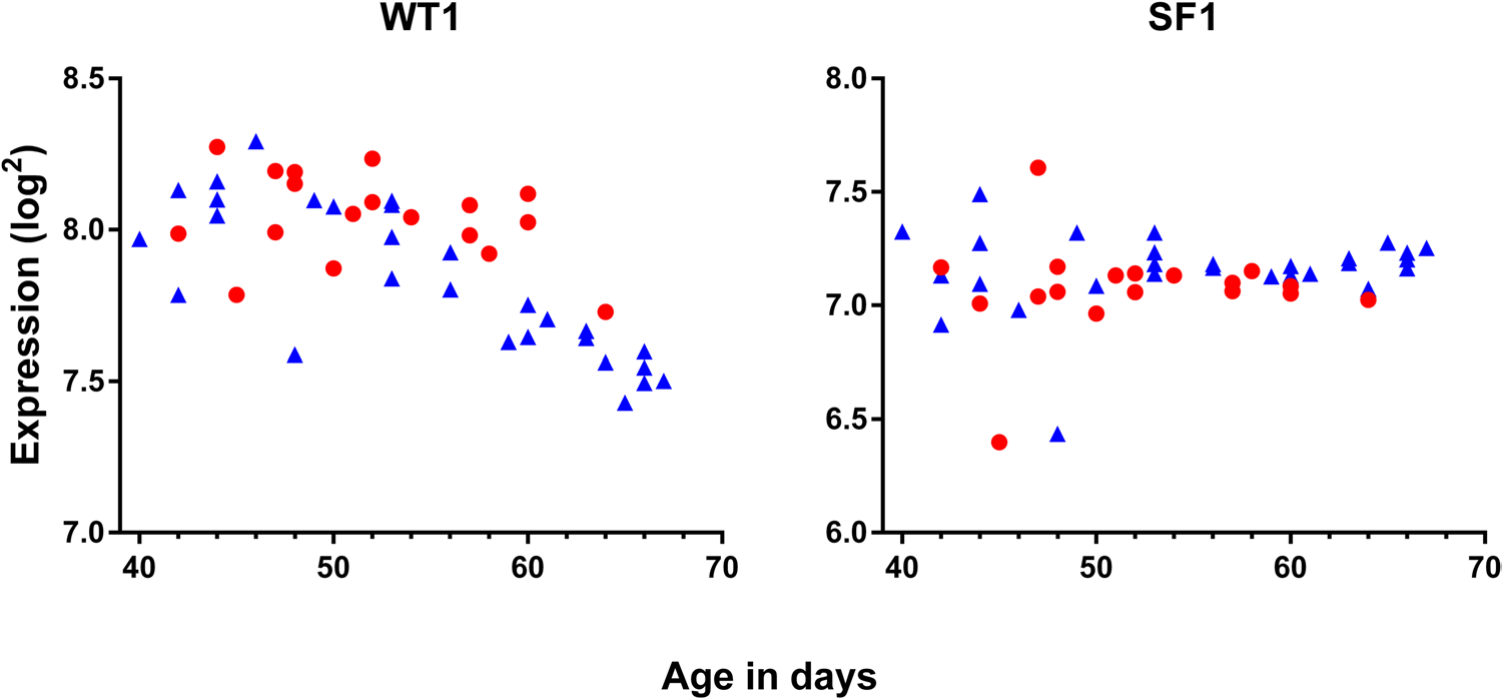
Gene expression of *WT1* and *SF1* necessary for the development of the bi-potential gonad prior to sex-differentiation in male and female gonads aged 40–68 days pc. Red represents female, blue represent males.

*WT1* and *SF1* were highly expressed in gonads irrespective of sex. The *WT1* gene was continuously expressed in ovaries whereas expression in testis decreased significantly over time (Fig. 1, Table 2). In contrast, *SF1* was expressed at a constant level irrespective of sex and age (Fig. 1, Table 2).

**Table 2:** Statistical analysis of gene expression patterns: Expression differences between sexes and correlation with age.

**Gene**	**Exp. difference female vs. male (p-value)** ***Linear model***	**Gender**	**Correlation with age (p-value)** ***Spearman***
**Bipotential gonad**			
*WT1*	Yes (0.0227)*	Female	No (>0.1)
		Male	Yes (<0.0001)
*SF1*	No (>0.1)	Female	No (>0.1)
		Male	No (>0.1)
**Sex differentiation**			
*SRY*	Yes (<0.0001)	Female	NA
		Male	Yes (<0.0143)
*SOX8*	No (>0.1)	Female	No (>0.1)
		Male	No (>0.1)
*SOX9*	Yes (<0.0001)*	Female	Yes (0.0155)
		Male	No (0.0689)
*SOX10*	No (>0.1)	Female	No (>0.1)
		Male	No (>0.1)
*SOX17*	No (>0.1)	Female	No (>0.1)
		Male	No (>0.1)
*GLI1*	Yes (<0.0001)	Female	Yes (0.011)
		Male	Yes (0.0002)
*PTCH1*	Yes (<0.0001)	Female	No (>0.1)
		Male	Yes (0.0072)
*AMH*	Yes (<0.0001)	Female	No (>0.1)
		Male	Yes (0.0014)
*AMHR2*	Yes (<0.0167)*	Female	No (>0.1)
		Male	No (>0.1)
*ETV5*	Yes (<0.0001)	Female	Yes (<0.0001)
		Male	Yes (0.0295)
*WNT2B*	Yes (<0.0001)	Female	Yes (<0.0001)
		Male	No (>0.1)
*WNT4*	No (>0.1)	Female	No (<0.056)
		Male	No (>0.1)
*RSPO1*	Yes (<0.0001)	Female	No (>0.1)
		Male	Yes (0.0007)
*FOXL2*	Yes (<0.0001)	Female	No (>0.1)
		Male	No (>0.1)
**Germ cells**			
*KIT*	Yes (<0.0001)	Female	Yes (<0.0001)
		Male	Yes (<0.0001)
*KITLG*	Yes (<0.0001)	Female	Yes (0.0025)
		Male	Yes (>0.0001)
*OCT-4*	Yes (<0.0001)*	Female	Yes (<0.0002)
		Male	Yes (0.0103)
*LIN28*	Yes (<0.0001)*	Female	Yes (0.0169)
		Male	No (>0.1)
*GAGE10*	No (>0.1)	Female	No (>0.1)
		Male	Yes (0.0003)
*GAGE12B*	No (>0.1)	Female	No (>0.1)
		Male	Yes (<0.0001)
**Steroidogenesis**			
*LHCGR*	Yes (<0.0001)	Female	No (>0.1)
		Male	Yes (0.0001)
*POR*	Yes (<0.0001)	Female	No (>0.1)
		Male	Yes (<0.0001)
*STAR*	Yes (<0.0001)	Female	No (>0.1)
		Male	Yes (<0.0001)
*CYP11A1*	Yes (<0.0001)	Female	No (0.0503)
		Male	Yes (<0.0001)
*CYP17A1*	Yes (<0.0001)	Female	Yes (0.0379)
		Male	Yes (<0.0001)
*HSD3β1*	Yes (<0.02)	Female	No (>0.1)
		Male	No (>0.1)
*HSD3β2*	Yes (<0.0001)	Female	No (0.0869)
		Male	Yes (0.0045)
*HSD17β3*	Yes (<0.0001)*	Female	No (0.0700)
		Male	Yes (<0.0001)
*HSD17β7*	Yes (<0.0001)	Female	No (>0.1)
		Male	Yes (<0.0001)
*ER-α/β*	No (>0.1)	Female	No (>0.1)
		Male	No (>0.1)

*Quadric model fitted data better than linear and was used.

### Expression of SRY and SOX

**Figure 2:**
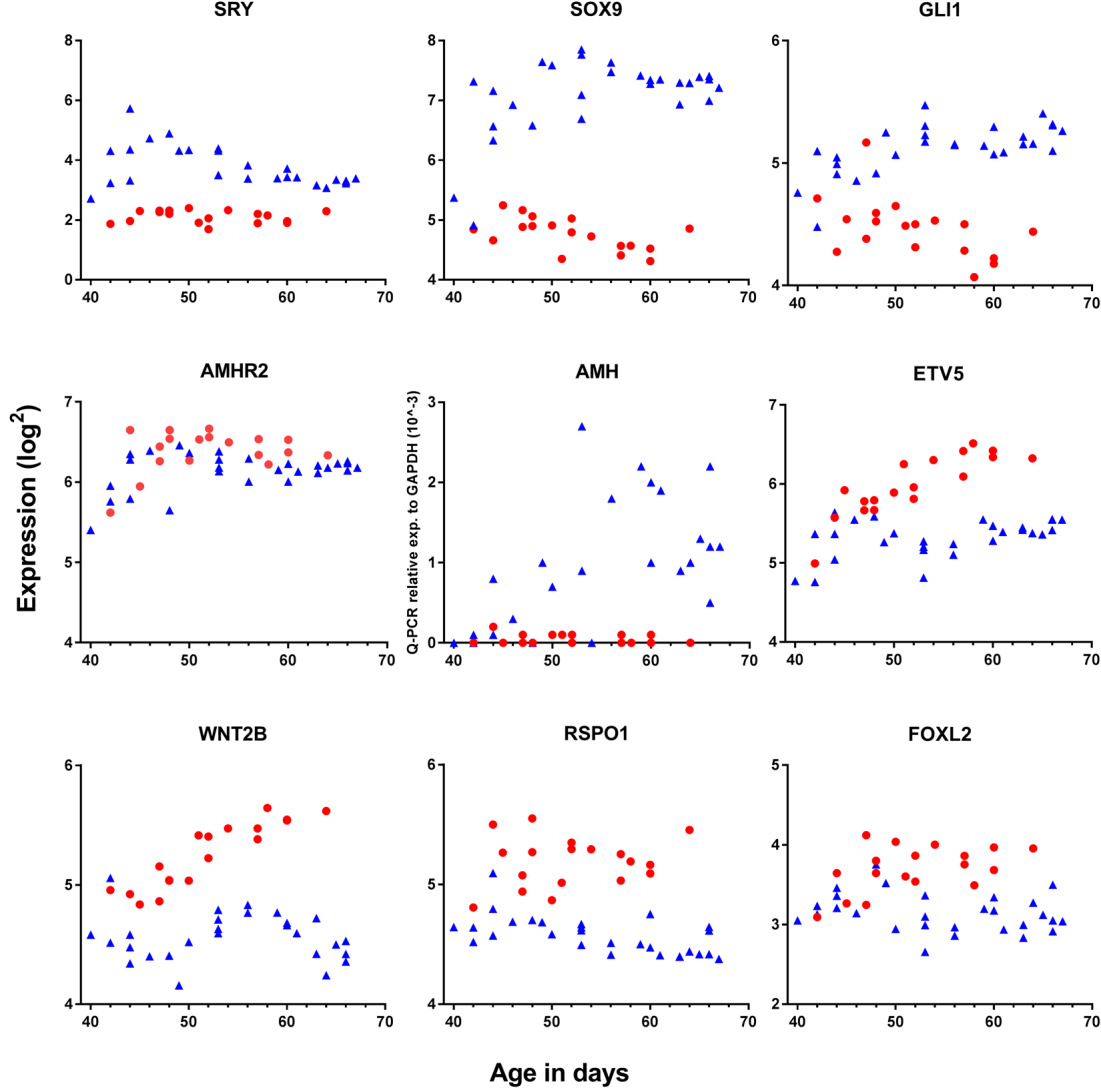
Expression of key genes involved in sex differentiation together with new potentially important genes (i.e. *GLI1, ETV5, WNT2B*) in male and female gonads aged 40–68 days pc. Red represents female, blue represent males.

**Figure 3:**
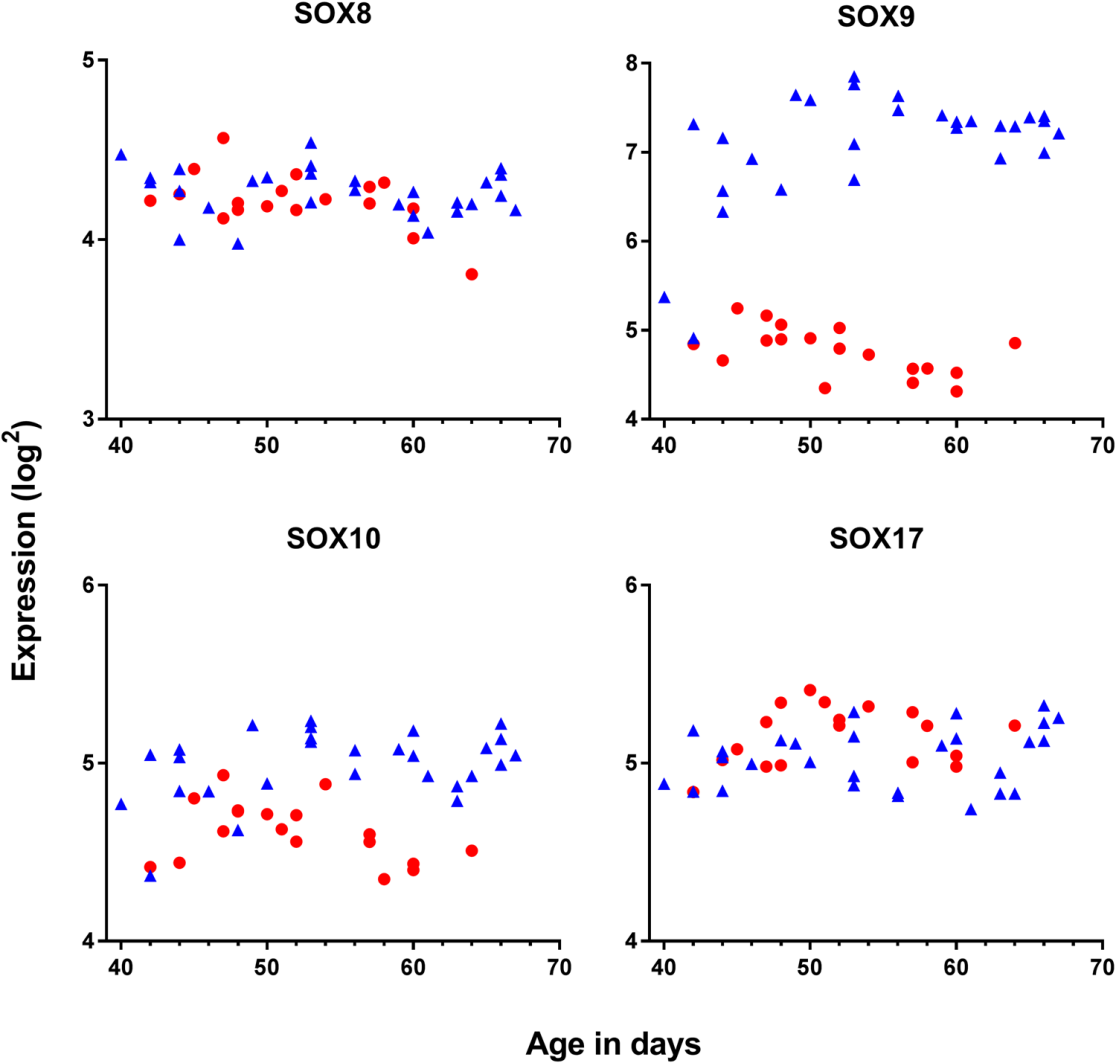
Gene expression of *SOX* genes in testis (blue) and ovaries (red) aged 40–68 days pc. *SOX9* was expressed significantly higher in testis than ovaries with no sex dimorphic expression in the *SOX8, SOX10*, and *SOX17*.

In testis, *SRY* expression peaked around day 44 pc where after expression steadily decreased to base level around day 60 pc; *SRY* was not expressed in ovaries (Fig. 2, Table 2, Supp. Figure 2). In testis, *SOX9* gene expression increased to a plateau around day 48 and was expressed significantly higher in testis than ovary (p < 0.0001); median *SOX9* log2 expression in females and males: 4.8 and 7.3, respectively (Fig. 3, Table 2). In testis, *SOX10* was expressed at significantly lower levels than *SOX9* (p < 0.0001, median *SOX10* log2 expression: 5.0) and *SOX8* at even lower levels (median *SOX8* log2 expression: 4.3) (Fig. 3, Table 2). Interestingly, *SOX10* was significantly higher expressed in males than females (p < 0.0001, median *SOX10* log2 in females: 4.6), whereas *SOX17* expression was higher in females than males (p < 0.0344, median *SOX17* log2 expression in females and males: 5.2 and 5.0, respectively) (Fig. 3, Table 2).

### Expression of new sex dimorphic genes (*GLI1, PTCH1, WNT2B* and *ETV5*)

During mammalian embryogenesis Hedgehog (Hh) signalling regulates cell differentiation and is in mice important for Leydig cell differentiation^51^. Gene expression of the Hh receptor protein patched homolog 1 (*PTCH1*) and the transcription factor glioma-associated oncogene homologue (*GLI1)* was significantly higher expressed in testis than ovary and an inverse correlation with age was found for both males and females (Fig. 2, Table 2). The Hh ligands: Desert (*DHH*), Indian (*IHH*), and Sonic (*SHH*) were expressed at a constant level (range: log2 3.5–4.5) with no sex dimorphism (data not shown). Surprisingly, there was no significant difference between sexes in *WNT4* expression and no correlation with age (Table 2), while *WNT2B* was significantly higher expressed in ovary than in testis and showed a significant positive correlation with age (Fig. 2, Table 2). *RSPO1* and *FOXL2* were also expressed significantly higher in ovary compared to testis, and the expression of *RSPO1* was positively correlated with age (Fig. 2, Table 2). In the ovary, ETS Variant 5 *(ETV5)* expression was significantly higher than in testis and was positively correlated with age (Fig. 2, Table 2).

### Expression of AMH

The microarray probes which covered the *AMH* gene also included the microRNA Mir4321, therefore no valid gene expression data on *AMH* was available from the microarray data. Therefore qPCR was used to evaluate *AMH* expression, where the probe detected a unique region of *AMH* excluding the mir-region. The expression of *AMH* was evaluated in all 46 gonadal samples; of these samples one result failed and two were outliers for no obvious reason, which were excluded (Fig. 2). In testis, *AMH* was highly expressed and positively correlated with age, whereas in ovary no expression was detected (Fig. 2, Table 2). In contrast, there was higher expression of *AMHR* in ovary than testis, with no correlation with age of either sex (Fig. 2, Table 2).

### Expression of germ cell marker genes

**Figure 4:**
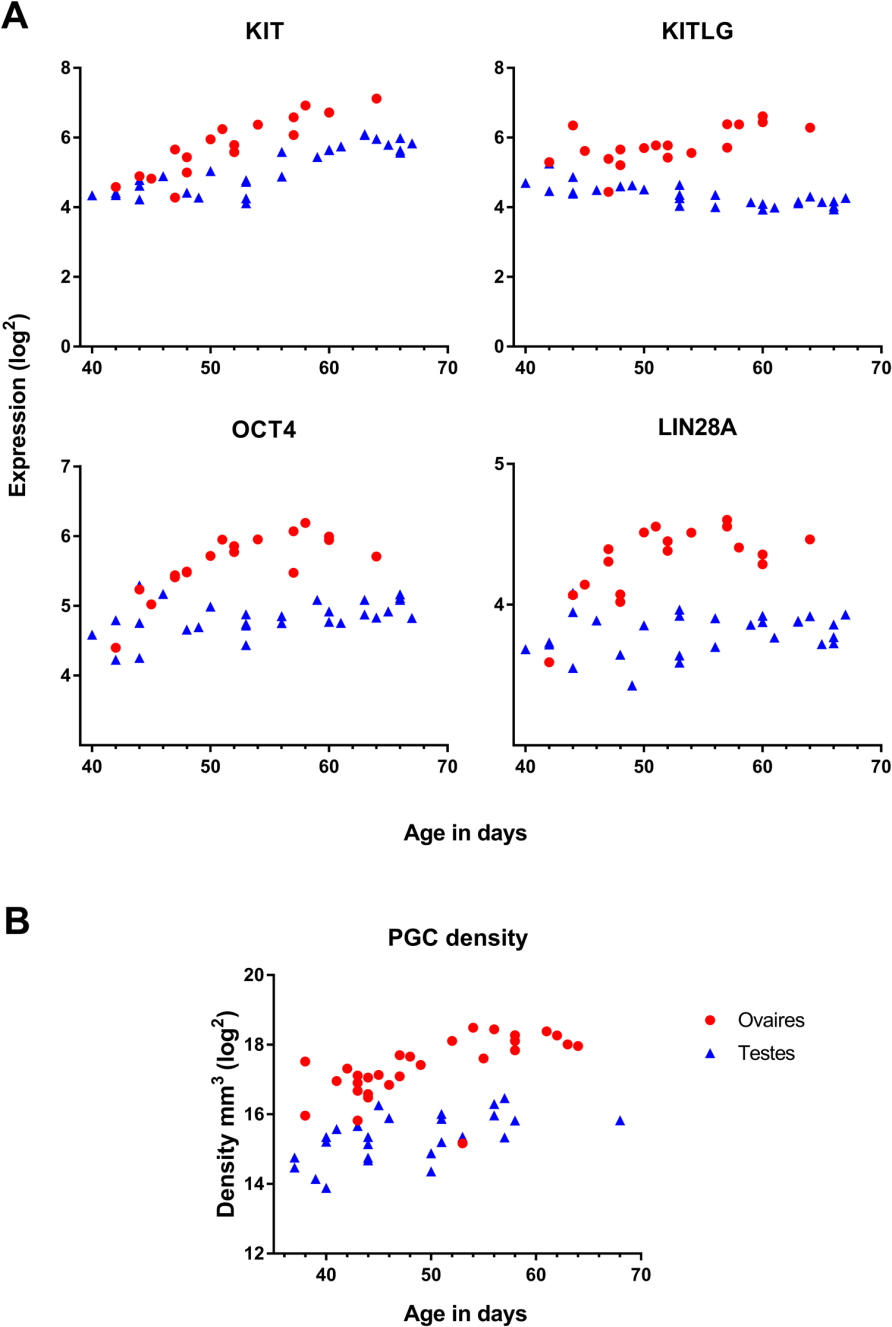
Gene expression of germ cell markers in testis and ovaries aged 40–68 days pc (**A**). The primordial germ cells (PGCs) density (i.e. PGCs per mm^3^) in testis and ovaries aged 35–68 days pc (**B**). Note the expression pattern of *OCT4* and *LIN28A* reflect the PGC density pattern. Red represents female, blue represent males.

The specific germ cell markers tyrosine-protein kinase (*KIT)*, octamer-binding transcription factor 4 *(OCT4)* and *LIN28*, together with the somatic-expressed KIT ligand (*KITLG*), was significantly higher expressed in ovary compared to testis and a positive correlation with age was found for KIT, KITLG, and OCT4 for both sexes (Fig. 4A, Table 2). The expression of the cancer/testis antigens *GAGE10* and *GAGE12B* was significantly positively correlated with age in testis but no correlation was found in ovary, and there was no significant difference in expression between sexes (Table 2).

### Germ cell density

Germ cell density (germ cells per mm^3^) was calculated from our previously published studies (29 females, 26 males)^8,9^, demonstrating a significantly higher germ cell density (p < 0.0001, R^2^ = 0.6403) in females than in males. A Spearman correlation test found a significant positive correlation between germ cell number and fetal age (females p < 0.0001, CI [0.725–0.939]; males p < 0.0001, CI [0.767–0.956]) and between germ cell density and fetal age (females p < 0.0001, CI [0.462–0.859]; males p < 0.0009, CI [0.284–0.812]). The pattern for each sex fitted a linear model better than a quadric model (Fig. 4B). The germ cell density pattern mirrored the mRNA expression pattern for KIT, OCT4, and LIN28A, confirming these genes are reliable germ cell markers (Fig. 4A,B).

### Expression of genes essential in steroidogenesis

**Figure 5:**
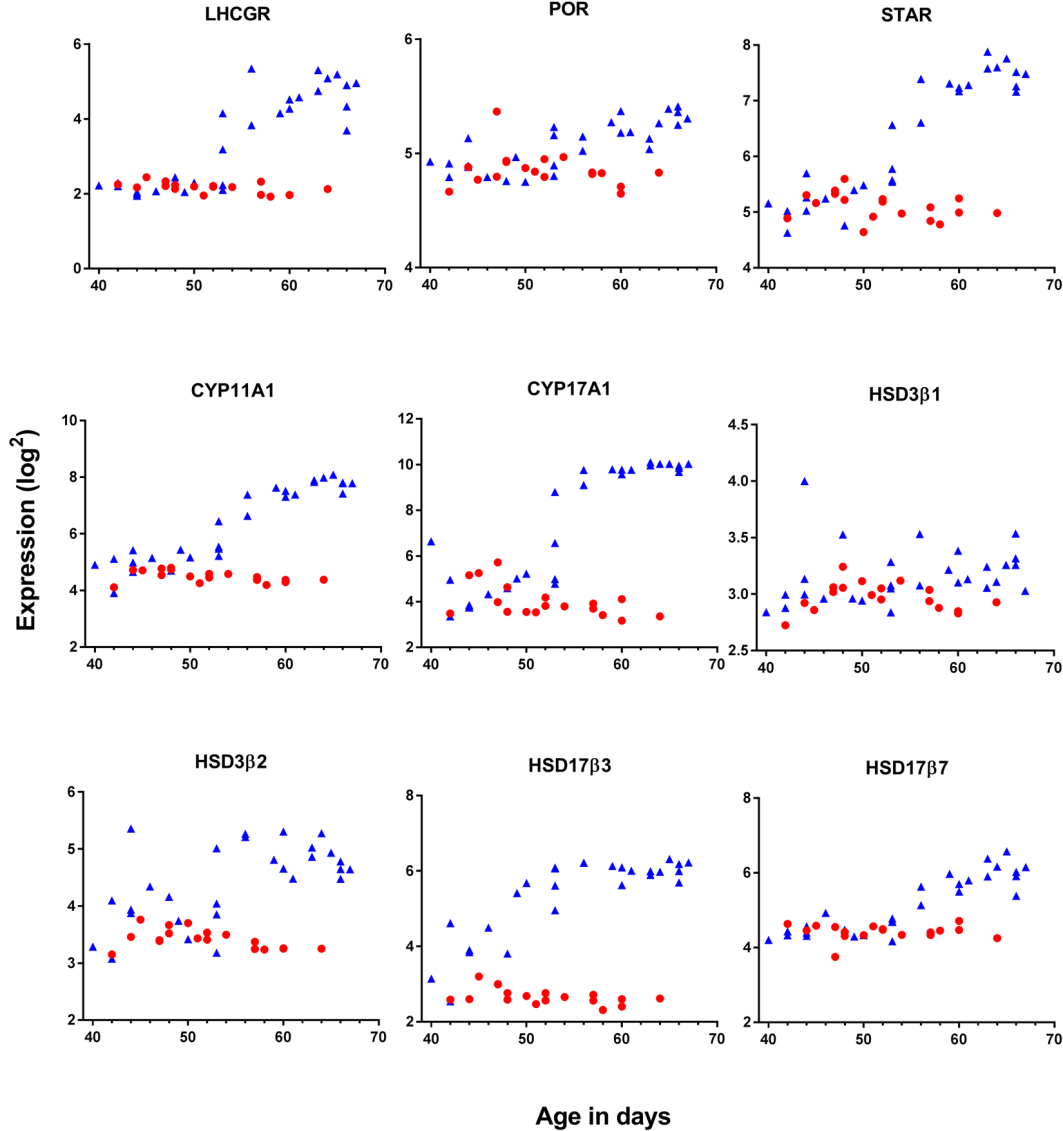
Gene expression of steroidogenic factors in testis and ovaries aged 40–68 days pc. All presented genes were significantly higher expressed in testis than ovaries. Further, a characteristic increase in expression was seen in testis around day 53 pc for several genes. Red represents females, blue represent males.

The steroidogenic genes *POR, STAR, CYP11A1, CYP17A1*, *HSD3β*2*, HSD17β3, HSD17β7*, and *LHCGR* were all expressed at higher levels in testis compared to ovary and all showed a characteristic increase in expression in testis around day 53 pc while no increase was observed in the ovary (Fig. 5, Table 2). *HSD3β1* was hardly expressed in either sexes (mean log2 exp < 3.1 for both), whereas *HSD3β2* expression was significantly higher in testis than in ovary (Fig. 5, Table 2). The array was able to identify 11 isoforms of *HSD17β*, and expression of *HSD17β1/β4/β8/β10/β11/β12* was confirmed (log2: 4.5–7.0) in both testis and ovary. *HSD17β3* and *HSD17β7* were more abundantly expressed in testis as compared to ovary (Table 2), while *HSD17- β2/β6/β13* was not expressed (data not shown). A simple linear – or quadric model described the data statistically equally well.

### Expression of the estrogen receptors ERα and ERβ

The estrogen receptors *α* and *β* (*ERα* and *ERβ)* were continuously equally expressed in gonads of both sexes with *ERα* being significantly higher expressed than *ERβ* (mean log2 expression *ERα:* 5.0 and *ERβ:* 3.0) with no significant difference between sexes and no correlation with age (Table 2).

### No effect of maternal smoking on embryonic and fetal gonadal gene expression

Half of both male and female gonads originated from mothers who smoked during their pregnancy (Supp. Table 1). To evaluate possible confounding effects of maternal lifestyle on gene expression, differences between smokers and non-smokers with respect to other lifestyle parameters were examined; however, there were no significant differences (Supp. Table 2). Further, there was no significant change in the expression (defined as a fold change ≥1.5) of any annotated genes (p > 0.05) between gonads prenatally exposed to maternal cigarette smoke and non-exposed gonads irrespective of sex.

### Confirmation of array data with qPCR

In 13 cases (6 females and 7 males) both fetal gonads were obtained. In these cases one gonad was included in the microarray analysis and one used for validation by qPCR analysis. The relative mRNA expression of *SRY*, *SOX9, STAR, CYP11A1*, and *LIN28A* were analyzed by qPCR, confirming the expression pattern found in the microarray analysis, with significantly different expression of *SRY, SOX9*, and *CYP11A1 LIN28* between sexes and borderline significant different expression of *STAR* (Sup. Figure 2). Data are presented as mean value of duplicate measurements including standard error of means.

### Expression cut-off

The cut-off for valid log2 intensities was determined by the qPCR analysis of the transcription factor *SRY* on the Y chromosome, which is expected not to be present in females. By microarray analysis *SRY* was detected at relatively low intensities: testis (median log2: 3.4) and ovaries (median log2: 2.1), which reflect a fold change of 2.51. The cut-off intensity for the microarray, validated by the qPCR results, was therefore defined as log2 = 2.1. In the microarray relatively low log2 *SRY* intensity was seen in testis, which can be attributed to the fact that entire gonads were included – not specific cell types – which may dilute the measured expression intensity. QPCR analysis in relation to endogenous *GAPDH* confirmed the results obtained by microarray (Supp. Figure 2).

### IHC staining

#### GAGE and LIN28 protein expression in germ cells

**Figure 6:**
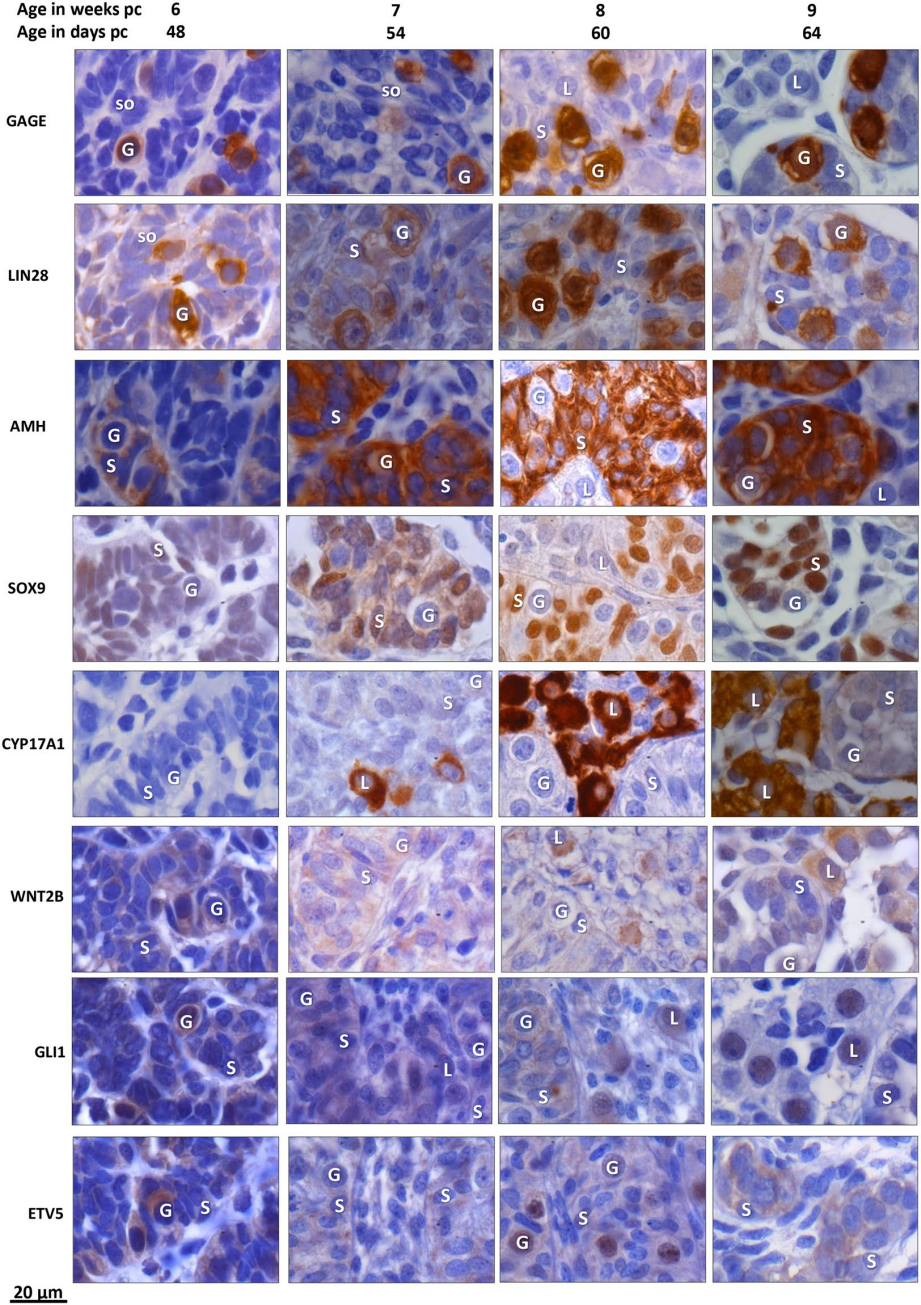
Immunohistochemical detection of prominent markers in human embryonic and fetal testis aged 48–64 days pc (week 6–9 pc). GAGE and LIN28 were detected in germ cells. AMH and SOX9 were detected in Sertoli cells. CYP17A1 was detected in Leydig cells from day 54 pc. WNT2B was detected in Sertoli cells in week 6–7 changing to Leydig cells in week 8–9. GLI1 was detected in testicular germ cells, Sertoli and Leydig cells. ETV5 showed a relatively low general expression in testis, with germ cells staining more intense than somatic cells. G = germ cell; S = Sertoli cells, L = Leydig cells; so = somatic cells.

**Figure 7:**
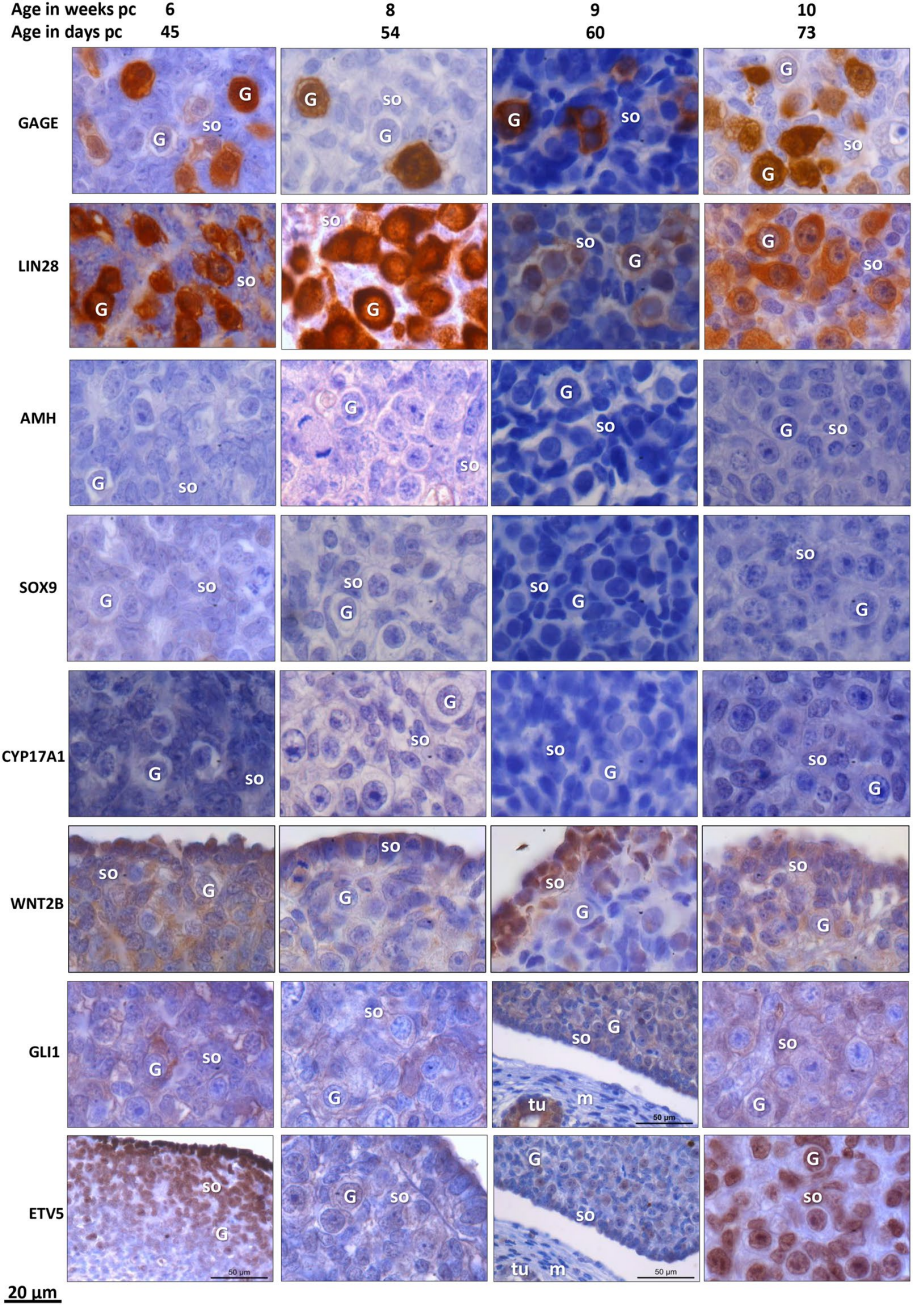
Immunohistochemical detection of specific markers in human embryonic and fetal ovary aged 45–73 days pc (week 6–10 pc). GAGE and LIN28 were detected in germ cells. AMH, SOX9, and CYP17A1 were not detected in ovaries. WNT2B was detected in ovarian somatic cells with an accumulation to epithelial cells and a faction of germ cells. GLI1 was generally expressed in the ovary and in the mesonephritic tublues without detectin in the mesenchymal stroma. ETV5 showed a relatively low general expression in ovary, with germ cells staining more intense than somatic cells. In 6 weeks ovary EVT5 was compartmentalized with strong expression in the ovarian surface epithelium with a decreasing staining intensity towards the mesonephros. ETV5 was also detected in mesonephritic tubuli. G = germ cell; so = somatic cells, m = mesonephros, tu = tubuli.

GAGE was detected in the nucleus of a subset of the germ cells in gonads of both sexes across the age range 45–73 days pc (Figs 6 and 7). LIN28 was also detected in germ cells in gonads of both sexes aged 45–73 days pc, with a cytoplasmic distribution.

#### AMH and SOX9 proteins expressed in Sertoli cells

The SOX9 transcription factor was detected in Sertoli cell nuclei, and AMH was detected in Sertoli cell cytoplasm in testes ages 48–64 days (Fig. 6). AMH and SOX9 were not detected in any ovaries (Fig. 7).

#### CYP17A1 expressed in Leydig cells

CYP17A1 was detected in some Leydig cells outside testicular cords from day 54, becoming more widely expressed in Leydig cells from testis aged 60–64 days (Fig. 6). No immunohistochemical staining of CYP17A1 was detected in any of the ovaries (Fig. 7).

#### WNT2B, GLI1, and ETV5 expressed in ovary and testis

In testes aged 48–54 days pc WNT2B, GLI1, and ETV5 was confined to testicular cords, whereas at later gestational age (day 60–64 pc) WNT2B and GLI1 was also detected in Leydig cells (Fig. 6). In contrast, ETV5 remained expressed within testis cords at day 60–64 with increasing intensity in germ cells (Fig. 6). In ovaries, WNT2B was detected in somatic cells particularly in ovarian surface epithelium between days 45–73 (Fig. 7). GLI1 and ETV5 were detected in mesonephritic tubuli and in the ovary with no staining in the mesenchymal stroma (Fig. 7). Further, at day 45 ETV5 showed a characteristic increase in staining intensity from medulla towards cortex and surface epithelium.

## Discussion

This is, to the best of our knowledge, the first study to provide a detailed mapping of the temporal expression of key regulatory genes governing sex-differentiation and initiation of steroidogenesis in the human embryonic and fetal gonad in both males and females. Additionally, new genes (i.e. *GLI1, ETV1* and *WNT2B)*, of potential importance in human sex-differentiation and development have been identified. The present study is, however, descriptive and the associations found between gene expression, sex and age may not reflect functional mechanisms.

In human testis sex-differentiation initiates as early as day 40–44 pc with expression of *SRY*, closely followed by expression of *SOX9*, while both are absent in ovaries. Previously, *SRY* expression has been detected at week 9 followed by a drop in week 11^46^, which corresponds to transient murine *Sry* gene expression at initiation of sex-differentiation^39,52,53^. The present data expand previous findings by advancing the onset of *SRY* expression to as early as day 40 pc (5½ weeks), enforcing its prominent role in initiation of sex-differentiation. The expression of *SRY* (median log2: 3.4) was relatively low compared to other evaluated genes, probably reflecting that *SRY* serves as a transcription factor where only little activity is sufficient to initiate the downstream signalling and is only expressed in Sertoli cells. Expression of *SOX9* was detected at both gene and protein level from day 48 pc, advancing the previously reported onset with approximately one week^46,54^. Collectively this suggests that human sex-differentiation start around one week earlier than previously described.

The gene expression pattern of fetal mice gonads during sex differentiation determined by microarray analysis resemble the patterns of the present study^55^: The undifferentiated gonad (11.5 dpc in mouse and 44 dpc in human) express a number of genes with similar expression in both sexes. Sex differentiation is initiated with the expression of *SRY* and *SOX9* (mouse 12.5 dpc, human 44–48 dpc) followed by expression of *AMH* (mouse 16.5 dpc, human 50–60 dpc) and the genes involved in the steroidogenesis (mouse 14.5–18.5 dpc, human 54 dpc). In mouse, genes involved in ovarian meiosis are expressed already at day 14.5^55^ whereas the human ovary do not initiate meiosis before week 10 pc^56^, which relatively is later than mice and beyond the evaluated period of the present study.

*Sox9* targets *Etv5*, which is expressed by Sertoli cells and play a role in maintaining the spermatogonial stem cell niche in fetal mice testis, but not in fetal ovary^57,58^. In neonatal mice Etv5 is also expressed in germ cells and is essential for spermatogonial proliferation^58^. In the adult mouse ovary Etv5 protein are localized to the granulosa, further females deficient for Etv5 are infertile suggesting Etv5 to be essential for the ovarian function later in life^59,60^. The present study found *ETV5* gene expression in both sexes with significantly higher expression in ovary, suggesting an earlier ovarian role in human than mice. At the protein level ETV5 showed a relatively low general expression in both testis and ovary, with testis staining both germ cells and Sertoli cells as seen in mice. Surprisingly, germ cells of both sexes stained more intense than somatic cells, suggesting that in humans ETV5 may play a direct role in early germ cell survival within the germ cell niece in both sexes, though further studies is needed to clarify this.

The present study found Hedgehog gene expression of the *PTCH1* receptor and the transcription factor *GLI1* in both sexes with significantly higher expression in testis than ovary. This sexual dimorphic expression of *Ptch1* and *Gli1* has previously been reported in fetal mice models^51,61,62^ with *Gli1* and *ptch1* being present fetal testis^63^ but absent in fetal ovary until the time of birth^64^. In mice, the dimorphic expression may reflect that *Gli1* is essential for Leydig cell differentiation in the early fetal testis but are not essential in the ovary before after birth when theca cells are established^63,64^. In fetal mice testis, it has been shown that Sertoli cell-derived Hh-signalling induce an activation of the interstitial cells, which becomes *Gli1* positive and may act as progenitors to two cell lines: 1) Steroid producing fetal Leydig cells and 2) non-steroidogenic progenitor cells, which differentiate into adult Leydig cells^63^. In the neonatal mouse ovary gene expression of *Ptch1* and *Gli1* has been detected in granulosa and theca cells of primary follicles – not in primordial, suggesting a role of Hh signalling in the communication between granulosa cells and theca cells in growing follicles^65,66^, a role in proliferation and androstenedione production has also been suggested^66,67^. The present study detect GLI1 in both testis and ovaries, with more intense staining in Leydig cells, confirming a potential role for GLI1 in human fetal Leydig cell differentiation as seen in mice. In contrast to mice, GLI1 protein is detected in the human fetal ovary suggesting that Hh mediated somatic cell communication initiates already in fetal life in humans, which is first seen after birth in mice. The first follicles occur in humans already in fetal life whereas in mice follicle formation is not seen until after birth^68^ which may support the suggestion of a general earlier activity and cell-cell-communication in the human ovary. The general staining of both ETV5 and GLI1 was specific to the gonad and mesonephritic tubuli with no staining in the mesenchymal stroma, suggesting these proteins may play a role in the early gonadal development of both sexes though a specific role cannot be pinpointed. It would have been fortunate to have qPCR analysis on all new genes; however this was not possible due to limited access to human embryonic and fetal material. The expressions of genes included in qPCR analysis were in good agreement with the expression detected by microarray analysis, suggesting compliance between the analysis methods.

Sertoli cells expressed detectable levels of AMH from day 44 pc, both detected by IHC and gene expression assays. As expected, AMH was undetectable in ovary, but surprisingly *AMHR2* was highly expressed in both sexes.

The present study identified onset of human testicular steroidogenesis to take place around day 53 pc (week 7½), with the key steroidogenic enzymes showing synchronous and very marked increase in gene expression. This was further supported by IHC-detection of CYP17A1-producing Leydig cells from day 54 pc and by qPCR analysis of *STAR* and *CYP11A1*. Testosterone synthesis has previously been measured in fetal testes from week 10 pc and onwards^69^ and the present study therefore advances initiation of steroidogenesis in testis by approximately two weeks. Elevated expression of some steroidogenic enzymes in fetal testis compared to ovary aged 9–20 weeks pc has been described^46^. Since the microarray data is based on gene expression in all cells of the entire gonad it is noteworthy that the first steroid-producing Leydig cells detected at day 54 contribute sufficiently for detection among the total gonadal mRNA pool. Expression of steroidogenic genes was low in the ovary, irrespective of age.

The family of HSD17β enzymes catalyze the conversion of 17-keto/hydroxyl steroids. We found high expression of 6 *HSD17β* -isoforms in gonads of both sexes, while *HSD17β3* and *HSD17β7* were more highly expressed in testis than ovary, suggesting that these isotypes may be important for early male steroidogenesis. In mice testis, *Hsd17β1* and *hsd17β3* are expressed already in fetal life at high levels^70^, while in rats they are only expressed in adulthood (reviewed by Griswold and Behringer, 2009). In the present study, *HSD17β3* is suggested the dominant isotype with an expression significantly higher in testis than ovary, whereas the *HSD17β1* is less expressed with no difference between sexes. *HSD17β3* has previously been detected in human fetal testis aged 9–20 weeks^46^ and has also been suggested the dominant isotype in adult human testis^71,72^. In embryonic and fetal testis, the regulation of HSD17β may differ between rodents and humans. Furthermore, we found the LH/chorionic gonadotrophin receptor (*LHCGR*) mRNA to be expressed in the human fetal testis from day 53, which extent findings by Macdonald and collegues^73^, who did not detect LHCGR protein until 12 gestational weeks and co-localization with HSD3β was not seen before 20 weeks’ gestation, suggesting that in first trimester steroidogenesis may not be mediated via LHCGR^73^.

Expression of aromatase (*CYP19A1*) was below detection limit in both sexes at these early developmental stages. Later in fetal life (week 14–22) aromatase has been detected in Sertoli, Leydig, and germ cells, but with no expression in week 35^74^. Estrogens have been suggested to block proliferation of the precursor Leydig cells^75^ and since proliferation of the testicular cells is high at gestational weeks 13–19^76^ it has been speculated that estrogens function in exactly this time frame as a regulator of precursor Leydig cell proliferation and differentiation, thereby affecting testosterone production^77^. Surprisingly, we find both *ERα* and *ERβ* to be continuously expressed in both sexes with expression of *ERα* being significantly higher than *ERβ*. This is in contrast to previous findings where *ERβ* but not *ERα* was detected in testes aged 14–22 weeks^74^. This may indicate a shift in estrogen receptor isoform expression in males from first to second trimester. In second trimester ovaries gene expression of both *ERα* and *ERβ* has been detected^45^. Taken together, steroidogenic enzymes are present in the embryonic male testis from day 53 pc both at mRNA and protein level advancing initiation of steroidogenesis with approximately one week, whereas aromatization does not initiate until later in fetal life, beyond the period evaluated in this study.

Among the key regulators of female sex-differentiation is RSPO1, an activator of the canonical WNT/β-catenin pathway opposing testis formation, with WNT4 as the key ligand^44,78^. Throughout the period of sex-differentiation significantly higher *RSPO1* expression was detected in ovary compared to testis^44^, confirming previous findings and supporting an important role in female sex-differentiation. WNT4 may also be a crucial female determinant: Wnt4 deficiency in female mice caused partial female-to-male sex reversal^79^ and inhibits endothelial and steroidogenic cell migration^80^, with disrupted initiation of meiosis^81^. Wnt4 may also have a testicular function in Sertoli cell organization and differentiation^80^. The present study demonstrates constitutive expression of *WNT4* from day 40–68 pc in the evaluated period irrespective of sex. Interestingly, a significantly higher level of *WNT2B* was detected in ovary compared to testis, suggesting a role of *WNT2B* in the human fetal ovary. Expression of WNT2B was by IHC also detected in somatic cells of testis and in ovaries with a stronger staining intensity in ovaries. Interestingly, WNT2B was compartmentalized with strong protein expression in the ovarian surface epithelium, a pattern which previously have been identified in the ovarian surface epithelium of adult rats^82^, and suggest a local regulation of tissue modelling in the ovary already in embryonic and fetal life. The characteristic increase in staining intensity towards the ovarian surface epithelium may suggest that WNT2B play a role in the survival and migration of the pre-granulosa cells, which have been suggested to originate from the ovarian surface epithelium wherefrom they populate the ovary^19,20^.

*FOXL2* is a highly conserved gene expressed during sex determination in pre-granulosa cells that later populate the ovarian medulla^83,84^. In mammals, FOXL2 can activate aromatase transcription during ovarian development and may prevent differentiation into testes^78^. Previous studies have detected *FOXL2* gene expression in human fetal ovaries aged 8–19 gestation with increasing expression from week 8 to 14^84^. The present study detected *FOXL2* gene expression in ovaries from around day 44 pc (week 6), exactly at the initiation of the ovarian sex differentiation. *FOXL2* was significantly higher expressed in ovaries than testis, suggesting that FOXL2 may be essential for proper differentiation of the human ovary.

Before sex differentiation, from day 40 pc, the embryonic gonads expressed high levels of *WT1* and *SF1. SF1* remained constant throughout the evaluated period with the expression of *WT1* being lower in testis compared to ovaries. The sex dimorphic expression of *WT1* may be due to a dilution factor. These genes have previously been described to play essential roles in early murine gonadal development and in development of kidneys and adrenal glands of both sexes^13,28,29,31,85,86^. In fetal male mice, *Sf1* expression persists in Leydig cells and in testicular cords after sex-differentiation indicating that *Sf1* may play a developmental role beyond the expression of steroidogenic enzymes^85^. In humans, heterozygous inactivating mutations in *SF1* have been associated with male to female sex reversal and adrenal failure, indicating that *SF1* is also essential for normal development in humans^87^.

The germ cell markers *KIT* (and its somatic-expressed ligand *KITLG*), *OCT4*, and *LIN28A* were more highly expressed in ovary compared to testis. This is consistent with the presence of more than twice as many germ cells in the ovary compared to the testis at 63 days pc^8,9,88^, confirming the validity of these markers as a surrogate for germ cell number over this developmental period. Interestingly, in testis expression of *KITLG* decreased over time while that of the *KIT* receptor continued to increase. KIT receptor has been detected in a fraction of testicular germ cells aged 7–17 weeks^89^.

Maternal cigarette smoking had previously been reported to cause a negative effect on the number of germ cells in first trimester embryos and fetuses^8^, an effect that persists through the second trimester^90^. We were unable to reveal any difference in gene expression between smoke exposed and non-exposed embryos and fetuses, which may reflect that gonads from smokers overall contain fewer cells but with the same relative contribution of different cell types compared to non-smokers, or because the present study evaluated gene expression in entire gonads – not in specific cells types, which then may dilute out differences.

Collectively the present findings provide a detailed temporal roadmap of changes in expression of key genes in human fetal gonads during sex-differentiation. Testis differentiation initiates already day 40 pc with expression of *SRY*/*SOX9*, followed by expression of the steroidogenic genes at day 53 pc. *GLI1, ETV1*, and *WNT2B* are newly identified genes, which may play a role in early human gonadal development, though further functional studies are needed to elucidate their potential roles.

### Ethical approval

‘The Scientific Ethical Committee for the Capital Region’ [KF (01) 258206] gave approval for this study. All participants gave informed consent before taking part and have given written consent for their data being included in publications. We confirm that all methods were performed in accordance with the relevant guidelines and regulations.

## Electronic supplementary material

Supp. Informaion
