# Supplementary material for: Temporal expression pattern of genes during the period of sex differentiation in human embryonic gonads
Source: Sci Rep. 2017 Nov 21;7:15961. doi: 10.1038/s41598-017-15931-3 (PMC5698446; doi:10.1038/s41598-017-15931-3)
Supplement: Supplementary file 1 — Supp. Informaion [file 41598_2017_15931_MOESM1_ESM.pdf]

Linn S. Mamsen, Emil H. Ernst, Rehannah Borup, Agnete Larsen, Rasmus H. Olesen, Erik Ernst, Richard A. Anderson, Stine G. Kristensen, Claus Y. Andersen

Supplementary information

Temporal expression pattern of genes during the period of sex differentiation in human embryonic gonads

Supp. Table 1.

Age distribution of male and female gonads included in the global gene expression analysis

Age in days pc (Age in weeks pc)	40—48 (5-6)	49—55 (7)	56—62 (8)	63—69 (9)	Total
**Males**					
Non-smoke exposed	2	1	3	5	11
Smoke-exposed	6	5	2	3	16
**Females**					
Non-smoke exposed	3	3	5	0	11
Smoke-exposed	4	2	1	1	8
**Total**					46

Supp. Table 2. The 46 participating womens' answers to questionnaire and age of fetuses [Mean ±SEM (range)]

Characteristics	Non-smokers (n=21)	Smokers (n=25)	*p*-Value
Age (years)	27.4 ±1.9 (18-47)	25.1 ±1.2 (18-40)	>0.1
BMI	24.0 ±0.9 (18.1-32.4)	22.7 ±0.9 (17.4-33.8)	>0.1
Exercise (h/day)	1.6 ±0.4 (0-5)	0.6 ±0.3 (0-2)	0.0522
Soft drinks (dl/day)	0.5 ±0.3 (0-5)	0.8 ±0.4 (0-5)	>0.1
Soft drinks light (dl/day)	0.7 ±0.4 (0-5)	0.4 ±0.3 (0-5)	>0.1
Coffee (cups/day)	0.9 ±0.3 (0-3.5)	1.4 ±0.3 (0-4)	>0.1
Tea (cups/day)	0.9 ±0.3 (0-4)	1.5 ±0.3 (0-4)	>0.1
Smoking (cigarettes/day)	0	11 ±0.9 (3-23)	<0.0001
Side-stream smoke (h/day)	0.3 ±0.2 (0-4)	2.1 ±0.4 (0-4)	<0.001
Fetal age (days pc)	55 ±1.7 (42-66)	52 ±1.5 (40-67)	>0.1

None of the participants have been drinking alcohol or taken over-the-counter medicine during the present pregnancy.

Linn S. Mamsen, Emil H. Ernst, Rehannah Borup, Agnete Larsen, Rasmus H. Olesen, Erik Ernst, Richard A. Anderson, Stine G. Kristensen, Claus Y. Andersen

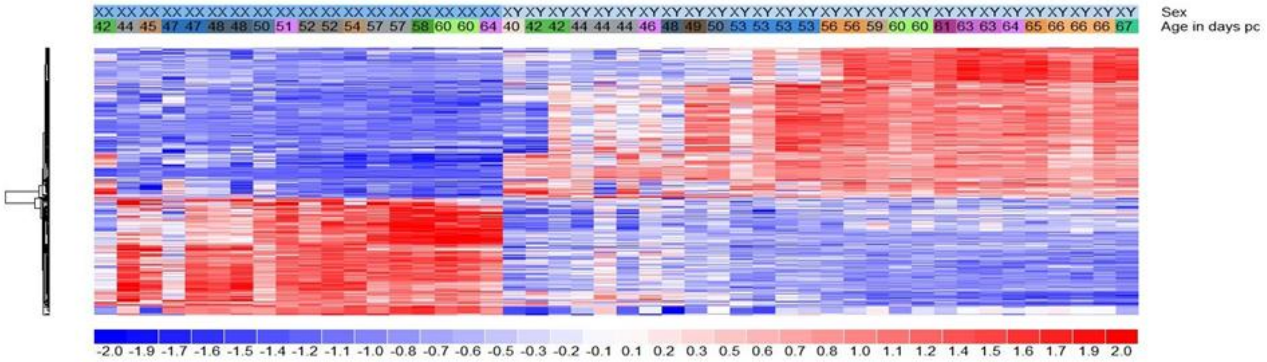

Supp. Fig. 1. The figure show a one way hierarchical clustering of the 319 probe sets that were differentially expressed between testis and ovaries. Gene expression values were standardized to mean of 0 and stdev of 1. Red pseudo color shows the standard deviation of expression above mean and blue pseudo color shows the standard deviation of expression below mean as indicated by the color bar. Samples are ranked according to age within each sex.

Linn S. Mamsen, Emil H. Ernst, Rehannah Borup, Agnete Larsen, Rasmus H. Olesen, Erik Ernst, Richard A. Anderson, Stine G. Kristensen, Claus Y. Andersen

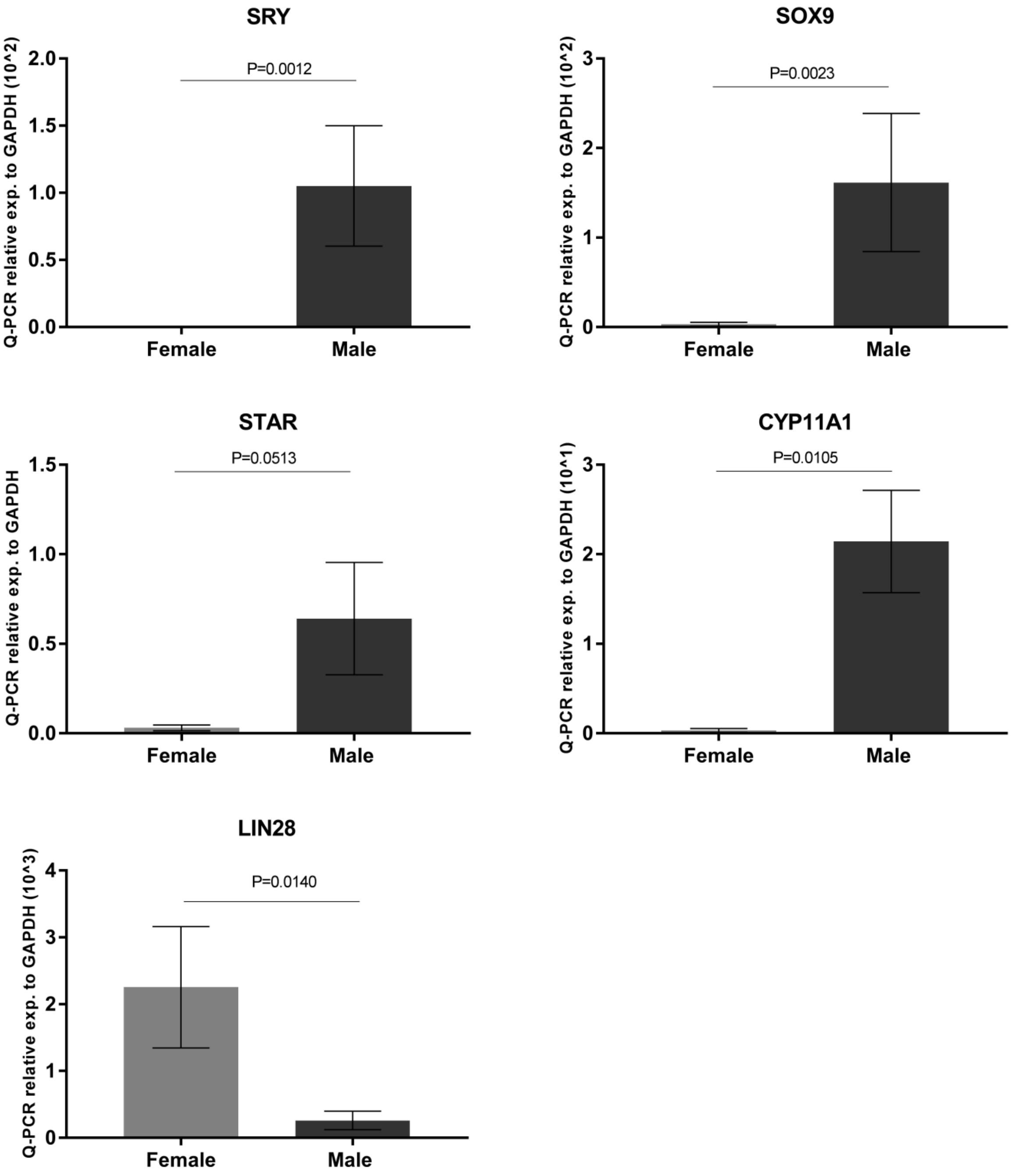

Supp. Fig. 2. Quantification of relative expression of SRY, SOX9, STAR, CYP11A1, and LIN28 transcripts by qPCR analysis. Expression levels were normalized to GAPDH. Presented data are mean values ±SEM in male (n=7) and female (n=6) gonads, with each sample contributing with a mean value of the duplicate measurements.
